# Ophthalmology

**Published:** 1900-09

**Authors:** Alvin A. Hubbell

**Affiliations:** Buffalo, N. Y.


### Progress in Medical Science.

### OPHTHALMOLOGY.

CONDUCTED BY ALVIN A. HUBBELL, M. D., Buffalo, N. Y.

### SPONTANEOUS ABSORPTION OF SENILE CATARACT IN THE INTACT CAPSULE.

DR. A. VON REUSS, of Vienna, (*Centralblatt für practische Augenheilkunde*, February, 1900; *Ophthalmic Review*, June, 1900,) finds that our knowledge of the conditions under which it is possible for spontaneous absorption of the cataractous lens to take place—a very rare occurrence, of course—is still meagre, and yet it is to a high degree desirable that we should know better for until we do so, we cannot tell whether it is possible that any attempt to deal with cataract without removal of the lens can ever be doomed to any result other than failure. Up to the present time some thirty or so of cases have been put on record. Reuss first of all describes two cases which he has himself seen, of which the first was published in 1885, but seems to have escaped the notice of all other writers on this topic. It is that of a man, E. L., whose right lens was extracted for cataract in 1868, obtaining vision = 20/20. In 1874, when he was sixty years of age, he suffered from an acute iritis, and was then observed to have a hypermature cataract in the left eye, but in 1876 the cataract had disappeared, except for a small sharply defined nucleus about 3mm. in diameter, lying at the level of the lower edge of the pupil. Above the pupil was quite black; vision = 20/50, and Jaeger 4, with +5 and +10d. respectively. In the operated eye there were a few vitreous opacities, and a small patch of choroiditis at the macula.

The more recent case was that of a man aged 75, whose left eye had undergone extraction for cataract in 1884; good sight had been obtained, but it had ultimately failed. The following year he had an attack of glaucoma in the right eye, which had yielded to poultices. He had a similar illness in 1898. About four months before Reuss wrote he began to find the vision in the right (unoperated) eye improving, and from p.l. it rose to 6/9 with +12, and J.3 with +16d. The a.c. was deep, the iris tremulous, the pupil black, there was distinct cupping of the disc, tension was normal, and the field of vision intact; close to the lower margin of the pupil a very fine transparent membrane could be faintly seen with a few white spots on it, but nothing one would call transparent capsule. Below this a small dark body could be seen, which was probably the shrunken nucleus. Although the patient admitted that on one occasion he had fallen and struck the back of his head, there was no real ground for supposing that the capsule had been ruptured; it appeared to be a genuine case of absorption within the intact capsule.

It used formerly to be believed that this was quite impossible, and Szili, who recorded one of the very early cases, felt himself called upon to suggest that as in his case the lens had swollen up very rapidly, the capsule might perhaps have ruptured spontaneously. It appears, too, that in a number of such cases the suspensory ligament gives way, and the shrivelled lens sinks downward; in the more recent of the two cases above recorded this had probably occurred. It is possible indeed that spontaneous luxation might precede absorption, but the comparatively healthy condition of the fundus and the possession of almost normal vision are distinctly in favor of any such assumption. In a certain number of the cases recorded by other writers also, it is to be observed that inflammation of one or another part of the uveal tract preceded the absorption, but what influence, if any, this can have upon such a result is not quite clear.

---

### DIONIN IN DISEASES OF THE EYE.

Dionin has been recently brought forward as an excellent general anodyne and hypnotic, whose use is not followed by unpleasant effects; and several observers have also found it beneficial in certain diseases of the eye. It is a compound known as the hydrochlorid of the mono-ethyl-ester of morphin (Gould's Year-book, 1900), and appears as a fine, white, crystalline powder, which is freely soluble in water. It is given internally in doses of one-fourth to three-fourths of a grain, and is said to stand in efficiency midway between morphin and codein.

Dr. Darier, of Paris, read a paper before the ophthalmological society of Paris in March, 1900, in which he detailed the excellent results obtained by means of dionin in various eye diseases. The first case cited was one of hyperacute, exceedingly painful iritis of rheumatic origin. In spite of repeated atropin instillations, the pupil had failed to enlarge, the eye remaining hyperemic and sensitive to light. A very minute quantity of dionin was then introduced into the conjunctival sac, when a severe burning sensation was experienced, and the patient complained of a sense of numbness and inability to move the eye. Two leeches were applied to the temples, and an hour later the pupil was found to be enlarged to its maximum extent, and the patient felt very much relief. Two days later the patient reported that not the slightest pain had been felt since, and the pupil, remained regularly and properly expanded. Similarly good results were obtained in several other like cases. In a case of double iridochoroiditis with complete posterior synechia, however, dionin did not afford such good analgesic action. In a case of double vascular keratitis with severe pain and extreme sensitiveness to light, the dionin caused at first an acute burning sensation and distinct chemosis, after which the pain and photophobia disappeared. Pustular keratitis in children was also treated, and, although the photophobia appeared to have been lessened, as the eyes could be opened better, yet it is difficut to render a proper decision in these cases.

In a case of acute parenchymatous keratitis the improvement was very rapid, and is ascribed as being largely due to the dionin employed. One application of dionin in a case of diffuse corneal infiltration, due to severe trauma, and resembling parenchymatous keratitis, sufficed to afford relief from the severe pains that had resisted other remedies and prevented sleep. A case of rheumatic episcleritis, in which sodium salicylate was ineffective, was treated with dionin with the result that, after the acute chemosis which first ensued, not only was the pain entirely relieved, but the episcleritis cured. Similarly good results in rapidly relieving pain were obtained in two cases of recurring corneal ulcers. Similar experiments were carried out in various ocular affections with other analgesics in order to determine their efficiency when compared with dionin. All those tested, however, were either far inferior to dionin, or caused such toxic symptoms (cold perspiration, vomiting, fainting spells, etc.,) as to preclude their employment. With dionin, on the other hand, no symptoms of this kind were observed. Dionin may be injected under the conjunctiva when it will also cause chemosis; and exert an analgesic effect. In conclusion, the author says that "dionin is, for the clinician, a powerful analgesic by means of which the most severe pains in iritis, iridocyclitis, ulcers, keratitis, and even glaucoma, may be reduced and relieved."

Dionin has also been employed recently by Dr. Wolffberg (*Therap. Monatsh.*, xiv., p. 237), of Breslau, in twenty-one cases of operations for cataract, because of the excellent results previously obtained in other eye diseases with the remedy. The number, though not very large, sufficed to enable the author to fully test it in this class of operations. It appeared from the treatment that though dionin does not seem to possess antiseptic properties, it nevertheless imparts a higher degree of resistance on the part of the corneal tissues toward any pathogenic microbes present than other remedies ordinarily employed. The increased flow of tears which occurs at first acts effectively in cleansing the eye; the ensuing conjunctival chemosis helps toward a better wound closure and more rapid restoration of the chamber; the swelling of the lids renders more difficult all unnecessary movement on their part and thus helps to render the dionin of more service in treatment without bandaging. The purely mechanical effect of the dionin-ophthalmia is moreover of considerable value in every form of bandage. In fact, the author states that he has never before experienced such a sense of security in treating the wounds following operations for cataract as since using dionin.

Colleagues who have seen cases a day or so after operations performed by the author have been convinced that the absence of all irritation, and the appearance of the wound, anterior chamber, and pupil, left nothing to be desired; and, further, that the application of dionin powder to the conjunctiva immediately after the operation was never found to be unpleasant because of sensitiveness.

Just as valuable as dionin has been found to be in all perforating corneal disturbances, just so indispensable is it also in superficial corneal wounds. The author also points out that in recurrent traumatic corneal erosions, which are so exceedingly painful, the use of dionin robs the disease of a large part of its terrors, because of the analgesic action exerted by the remedy. The dionin is more particularly useful in this disease, in which cocaine can not be used because of its deleterious effect on corneal epithelial formations. In combination with atropin, dionin is besides very useful in more rapidly producing mydriasis, at times even in iritis. Further, dionin is useful in glaucoma, particularly in chronic inflamed forms, when combined either with eserine or pilocarpin, or even both.—*Merck's Reports*.

---

### GLAUCOMATOUS IRITIS.

OCCASIONALLY a case of iritis (*Centralblatt für Augenheilkunde*, February, 1900; *Ophthalmic Review*, July, 1900,) occurs in which the tension is found to be increased and in which use of atropin is on this account attended with some risk of further evil, while if one withholds the mydriatic he is apt to be a mere spectator of the formation of disastrous synechiæ. Wagner, of Odessa, relates, at considerable length, an instance of such a condition. Briefly, it was that of a man who for a number of years had suffered yearly from acute iritis of one or other eye; the attacks were attended with much pain, with increased tension, and with copious, semi-opaque exudation into the anterior chamber; treatment of the ordinary type did not materially alter the course of the disease, and though for a long time each attack appears to have been entirely recovered from, yet in process of time as the recurrences multiplied vision had been distinctly degenerating. A paracentesis in one eye and an iridectomy in the other seemed to reduce the severity, if not the frequency, also of the successive attacks, and the use of a myotic, in spite of the risk of promoting unwelcome adhesions, undoubtedly not merely greatly relieved the acute symptoms, but also assisted largely to prevent recurrences.

